# Safety and efficacy of ciltacabtagene autoleucel for relapsed/refractory multiple myeloma: a CIBMTR study

**DOI:** 10.1038/s41408-026-01496-w

**Published:** 2026-04-14

**Authors:** Doris K. Hansen, Danai Dima, Hira Mian, Jakob Devos, Ruta Brazauskas, Temitope Oloyede, Aimaz Afrough, Nausheen Ahmed, Larry D. Anderson, Rahul Banerjee, Jesus G. Berdeja, Aram Bidikian, Binod Dhakal, Ajoy Dias, Yvonne Efebera, Muhammad Salman Faisal, Lohith Gowda, Hamza Hashmi, Abu-Sayeef Mirza, Meera Mohan, Ravi Narra, Ashley E. Rosko, Mark Schroeder, Taiga Nishihori, Heather Landau, Saad Usmani, Marcelo C. Pasquini, Othman S. Akhtar, Surbhi Sidana, Krina K. Patel

**Affiliations:** 1 H. Lee Moffitt Cancer Center & Research Institute, Tampa, FL USA; 2 University of Washington/Fred Hutchinson Cancer Center, Seattle, WA USA; 3 McMaster University/Juravinski Cancer Centre, Hamilton, ON Canada; 4 CIBMTR (Center for International Blood and Marrow Transplant Research), Milwaukee, WI USA; 5 Medical College of Wisconsin, Milwaukee, WI USA; 6 University of Kansas Medical Center, Kansas City, KS USA; 7 UT Southwestern Medical Center/Harold C. Simmons Comprehensive Cancer Center, Dallas, TX USA; 8 Tennessee Oncology, Nashville, TN USA; 9 Yale School of Medicine/Yale Cancer Center, New Haven, CT USA; 10 National Cancer Institute, Bethesda, MD USA; 11 OhioHealth, Columbus, OH USA; 12 OU Health Stephenson Cancer Center, Oklahoma City, OK USA; 13 Memorial Sloan Kettering Cancer Center, New York, NY USA; 14 Ohio State University Comprehensive Cancer Center – James Cancer Hospital, Columbus, OH USA; 15 Washington University School of Medicine/Siteman Cancer Center, St. Louis, MO USA; 16 Cleveland Clinic, Cleveland, OH USA; 17 Stanford University School of Medicine, Stanford, CA USA; 18 The University of Texas MD Anderson Cancer Center, Houston, TX USA

**Keywords:** Cancer therapy, Cancer immunotherapy

## Abstract

Ciltacabtagene autoleucel (cilta-cel), an anti-B-cell maturation antigen (BCMA) chimeric antigen receptor T-cell (CAR-T) therapy, was approved in 2022 for heavily pretreated relapsed/refractory multiple myeloma (RRMM). This study evaluates the safety and efficacy of cilta-cel in RRMM patients reported to the Center for International Blood and Marrow Transplant Research registry between March 2022 and December 2023 who met commercial release specifications. Among 595 patients, median age was 64 years, 57% were male, and 70% had ≥1 comorbidity. Extramedullary disease and marrow plasma cell burden ≥ 50% were present in 13% and 14% of patients, respectively. The median number of prior lines of therapy was 7 and 8% had received prior BCMA-directed therapy. Median follow-up was 12 months (range, 1–25 months). Cytokine release syndrome occurred in 80% (≥ grade 3: 4%) and immune effector cell–associated neurotoxicity syndrome (ICANS) in 22% (≥ grade 3: 4%). Non-ICANS neurotoxicity was seen in 5% (n = 31), including Parkinsonism in 2.7% (n = 16) and cranial nerve palsies in 2.5% (n = 15), primarily cranial nerve VII (n = 12/15). Infections occurred in 47% and treatment-related mortality was 5%. The best overall response rate was 87%, with ≥ very good partial response rate in 75%, and ≥ complete response rate in 35%. Estimated 12-month progression-free and overall survival were 73% (95% CI: 68–77%) and 85% (95% CI: 81–88%), respectively. This represents the largest standard-of-care (SOC) study of cilta-cel in RRMM patients to date. Despite advanced disease and high comorbidity burden, cilta-cel demonstrated favorable safety and efficacy, supporting its use in clinical practice.

## Introduction

B-cell maturation antigen (BCMA)-directed chimeric antigen receptor T-cell therapy (CAR-T) has revolutionized the treatment landscape of multiple myeloma [1–3]. In February 2022, the United States (US) Food and Drug Administration (FDA) approved ciltacabtagene autoleucel (cilta-cel), an anti-BCMA CAR-T with 2 identical camelid-derived heavy-chain antibodies on each chimeric receptor, for patients who have received ≥4 prior lines of therapy with subsequent updated earlier line approval in April 2024 [4–8]. Cilta-cel approval was based on the Phase Ib/II CARTITUDE-1 clinical trial demonstrating an overall response rate (ORR) of 98% and complete response or better (≥CR) of 83%. Median progression-free survival (PFS) was 34.9 months, and overall survival (OS) was 60.7 months [6, 8]. In terms of safety, cytokine release syndrome (CRS) and immune effector cell-associated neurotoxicity syndrome (ICANS) occurred in 95% and 17% of patients, respectively. Non-ICANS neurotoxicity (NINT), including Parkinsonism and cranial nerve palsies, occurred in 12% of patients [4–8].

Clinical trials have stringent eligibility criteria, which lead to the inclusion of patients with limited comorbidities and may not be reflective of routine clinical practice [9–14]. Utilizing the Center for International Blood and Marrow Transplant Research (CIBMTR) infrastructure and registry, we aimed to investigate the safety and efficacy of cilta-cel in a real-world patient population overall and in high-risk patient subgroups. We hypothesized that in standard-of-care (SOC), cilta-cel would demonstrate safety and efficacy profile similar to that of CARTITUDE-1.

## Methods

### Data source

CIBMTR is a research collaboration between the National Marrow Donor Program (NMDP) and the Medical College of Wisconsin. More than 375 medical centers worldwide submit clinical data to CIBMTR about hematopoietic cell transplant, CAR T-cell therapy, and other cellular therapies for treatment of malignancies. CIBMTR data undergoes a series of automated and manual quality checks to maximize its validity. In addition, CIBMTR audits each contributing center periodically. CIBMTR protects the privacy and human rights of participants and obeys international laws and ethical guidelines. The NMDP Institutional Board reviews CIBMTR research. Patients or guardians give informed consent to share data for research.

### Patients

All patients with relapsed/refractory multiple myeloma (RRMM) who received SOC cilta-cel after ≥4 prior lines of therapy from March 2022 to December 2023, and with at least a 100-day post CAR-T evaluation, were eligible for study inclusion. Patients receiving CAR-T who did not meet FDA release criteria for an in-specification product were excluded. High-risk cytogenetics was defined by the presence of *t*(4;14), *t*(14;16), *t*(14:20) and/or deletion 17p at any time before CAR-T infusion. Extramedullary disease (EMD) was reported as present if noted organ involvement as assessed by imaging (computed tomography, magnetic resonance or positron emission tomography) before CAR-T infusion as defined by each center, and may include both true extramedullary and paramedullary disease due to limitations in registry-level classification [15]. As part of the study was conducted during a U.S. fludarabine shortage, alternative lymphodepletion (LD) regimens were given at each center’s discretion to include bendamustine and cyclophosphamide.

### Outcomes

The primary outcomes were efficacy, defined as ORR and PFS from CAR-T infusion. Secondary endpoints included OS and other safety measures such as CRS, ICANS, non-ICANS NT, immune effector cell-associated hemophagocytic lymphohistiocytosis-like syndrome (IEC-HS), cytopenias, infections, second primary malignancies (SPM), and treatment-related mortality (TRM). CRS and ICANS were assessed per the American Society of Transplantation and Cellular Therapy consensus criteria [16], whereas myeloma responses were assessed per International Myeloma Working Group criteria [17]. Non-ICANS NT was defined based on the reporting of specific neurologic symptom patterns consistent with delayed or atypical neurotoxicity in the CIBMTR registry. Detailed longitudinal neurologic characterization and precise adjudication of onset timing were limited by the structure of the CIBMTR forms. Neutrophil recovery was defined as achieving a neutrophil count of >500/µL for 3 consecutive days with the first day counted as the day of recovery, while platelet recovery was defined as a platelet count of >20,000/µL without platelet transfusion within the last 7 days of the follow-up visit. Prolonged cytopenias were defined as absence of neutrophil and/or platelet recovery by 30 days following cilta-cel infusion. In line with CIBMTR definitions, infections were considered clinically significant if they required treatment. Episodes of neutropenic fever without an identifiable source and upper respiratory tract infections presumed viral but lacking pathogen confirmation were excluded. TRM was defined as death in the absence of disease progression.

### Statistical analysis

Distribution of patient characteristics were examined using chi-square or Fisher’s exact tests for categorical variables or Wilcoxon rank-sum test/Kruskal–Wallis test for continuous variables. Cox proportional hazards regression models were used to estimate hazard ratios (HR) and 95% confidence intervals (CI). Multivariable regression models were performed for the following outcomes: ≥grade 2 CRS, any grade ICANS, best ORR, ≥CR, PFS, and OS. A stepwise variable selection approach with a significance threshold of *P* < 0.05 was used to identify covariates for inclusion in the final models. Logistic regression was used for binary outcomes (≥grade 2 CRS, any grade ICANS, best ORR, ≥CR), whereas Cox proportional hazard models were applied for time-to-event endpoints (PFS, OS). The proportional hazards assumption and potential cross-variable interactions were tested on all models. Hazard ratios (HR) or odds ratios (OR) with 95% CI were estimated and reported for all models. Variables included in the models were (1) age category (<60, 60–69, and ≥70); (2) sex; (3) race (White, Black or African-American, others, not reported); (4) Eastern Cooperative Oncology Group performance score at infusion (ECOG 0-1, ECOG ≥ 2, missing); (5) number of prior lines of therapy (4–6, 7–10, >10); (6) International Staging System (ISS) stage at diagnosis (I, II, III, and unknown); (7) marrow plasma cell burden before infusion (<50%, ≥50%, unknown); (8) lactate dehydrogenase (LDH) before infusion (normal, elevated, unknown); (9) baseline ferritin (≥150 ng/mL vs <150 ng/mL); (10) presence of EMD; (11) prior BCMA-directed therapy; (12) disease status at infusion (≥very good partial response [VGPR], PR, stable disease, progressive disease, and unknown status); (13) type of lymphodepleting chemotherapy (fludarabine and cyclophosphamide vs bendamustine); (14) absolute neutrophil count before cilta-cel infusion (≥750/µL vs. <750/µL); (15) hemoglobin before cilta-cel infusion (≥8 g/dL vs <8 g/dL); (16) platelet count before cilta-cel infusion (>50,000 vs. <50,000/µL); and (17) cilta-cel dosing (≥0.7 million cells vs. < 0.7 million cells) and (17) hematopoietic cell transplantation-specific comorbidity index.

PFS was defined as the time from cilta-cel infusion until disease progression, death from any cause, or last follow-up. OS was defined as the time from cilta-cel infusion until death from any cause or last follow-up. The Kaplan–Meier method was used to estimate overall PFS and OS, and log-rank tests were used to compare PFS and OS by selected patient characteristics. All *P* values were 2-sided, and the difference between 2 variables was considered significant at *P* < 0.05.

#### Ethical approval

All Center for International Blood and Marrow Transplant Research studies are reviewed and approved by the National Marrow Donor Program Institutional Review Board, and all patients (or their legal guardians) provided informed consent for data collection and research.

## Results

### Patient characteristics

A total of 595 RRMM patients were infused with SOC cilta-cel (Supplementary Fig. 1). Table 1 shows the distribution of patient characteristics. The median age was 64 years (range: 34–84) and 57% were male. Most patients were White (77%) with Black and Hispanic patients representing 15% and 9% of the cohort, respectively. Most patients had an ECOG PS of 0–1 (89%). At least 1 clinically significant comorbidity was present in 70% of patients before cilta-cel. Common comorbidities included moderate to severe cardiac disease (26%), pulmonary disease (24%), renal impairment (5%), cerebrovascular disease (4%), and history of prior malignancies (15%).

**Table 1:** Baseline characteristics of patients who received standard-of-care ciltacabtagene autoleucel.

Characteristic	*N* (%) or median (range), *N* = 595
Age, years	64 years (34–84)
Age ≥70 years	134 (23)
Sex	
Male	340 (57)
Ethnicity	
Hispanic (any race)	54 (9)
Race	
White	456 (77)
Black or African American	89 (15)
Asian/Pacific Islander	20 (3)
More than 1 race	4 (1)
Unknown	26
ECOG PS	
0–1	527 (89)
2–4	19 (3)
Unknown	49
Myeloma subtype	
Oligo/Non-secretory	15 (3)
ISS disease stage	
I	154/260 (59)
II	66/260 (25)
III	40/260 (15)
Unknown	335
Cytogenetic abnormality	
Any high-risk cytogenetics^a^	142/530 (27)
High-risk cytogenetics including 1q	277/530 (52)
Unknown	65
High marrow burden BMPCs ≥ 50%	
Yes	54/373 (14)
Unknown	222
Extramedullary disease	
Yes	50/382 (13)
Unknown	213
Plasma cell leukemia (active or history)	6 (1)
Prior therapies	
Number of prior antimyeloma therapies	7 (4–24)
Prior autologous SCT	471 (79)
Prior allogeneic SCT	4 (1)
Prior anti-BCMA therapy	45 (8)
Prior belantamab mafodotin	37 (6)
Prior anti-BCMA CAR-T	3 (1)
Prior bispecific antibody	7 (1)
>1 BCMA targeting therapies^b^	1 (0.2)
Triple-exposed	507 (85)
Penta-exposed	328 (55)
Clinically significant comorbidity	416 (70)
Cardiac disease	81 (14)
Arrythmia	61 (10)
Heart valve disease	12 (2)
Cerebrovascular disease	25 (4)
Hepatic	6 (1)
Pulmonary	140 (24)
Renal	29 (5)
Prior malignancy	91 (15)
Baseline cytopenia	
Hemoglobin <8 g/dL	54 (9)
Platelets <50,000/μL	51 (9)
Absolute neutrophil count <750/μL	21 (4)
Baseline inflammatory markers	
Ferritin ≥150 ng/mL	172/328 (52)
CRP ≥ 0.5 mg/dL	158/353 (45)
Bridging therapy	339 (57)
Disease status prior to infusion	
PR or better	145 (24)
SD/PD	445 (75)
Unknown	5
Lymphodepletion chemotherapy	
Fludarabine + cyclophosphamide	461 (78)
Bendamustine	111 (19)
Other	22 (4)
Unknown	1
CAR T-cell dose ≥ 0.7 million	172 (29)
Days from leukapheresis to infusion	63 (56–71)

Cytopenias were defined as hemoglobin <8 g/dL, absolute neutrophil count <750/μL and platelets <50,000/μL. Percentage totals may exceed 100% due to rounding. When 10% of more data was missing for a given variable, denominator changed to patients with data available.

*BCMA* B-cell maturation antigen, *BMPC* bone marrow plasma cells, *CAR T-cell therapy* chimeric antigen receptor T-cell therapy, *CRP* C-reactive protein, *ECOG PS* Eastern Cooperative Oncology Group performance status, *ISS* international staging system, *PD* progressive disease, *PR* partial response, *SCT* stem cell transplantation, *SD* stable disease. ^a^High-risk cytogenetics: Includes del(17p), t(4;14) and t(14;16). High marrow burden was defined as > 50% plasma cells before CAR-T infusion; Penta-exposed disease: exposed to lenalidomide, pomalidomide, bortezomib, carfilzomib, and daratumumab or isatuximab. Triple-exposed disease: exposed to an IMiD, PI, and an anti-CD38 monoclonal antibody.

^b^Patient who received bispecific antibody and belantamab.

The median number of prior lines of therapy was 7 (range: 4–24), 85% of patients were triple-class exposed, and 55% were penta-drug exposed. High-risk cytogenetics was present in 27%, with advanced stage ISS II–III, EMD, and high tumor burden (≥50% bone marrow plasma cells) observed in 40%, 13%, and 14% of patients, respectively. Notably, 8% of patients had received prior BCMA-directed therapy, consisting of an antibody drug conjugate (ADC), belantamab mafodotin (6%, *n* = 37), prior CAR-T 0.5%, *n* = 3), bispecific antibody (teclistamab, 1%, *n* = 7), and more than 1 prior BCMA therapy in 1 patient who received both teclistamab and belantamab mafodotin (Supplementary Table 1).

Overall, 5 patients had received prior CAR-T, primarily through investigational trials, including bb21217 (*n* = 1), a CD28-based anti-BCMA CAR-T (*n* = 1), an unknown anti-BCMA CAR-T (*n* = 1), a clustered regularly interspaced short palindromic repeats–(CRISPR)-edited allogeneic anti-BCMA CAR-T (*n* = 1), and anti–G protein–coupled receptor, class C, group 5, member D (GPRC5D) CAR-T (*n* = 1). Bridging therapy was administered in 57% of patients (Supplementary Table 2), and 24% achieved a disease status of ≥ partial response (PR) prior to infusion. Lymphodepletion chemotherapy consisted of fludarabine and cyclophosphamide in 78%, bendamustine in 19% during the era of fludarabine shortage in the U.S., and other regimens in 4%. The median follow-up for this cohort was 12 months (range, 1.1–25.4 months).

### Safety

Any grade, grade ≥2, and grade ≥3 CRS occurred in 80%, 20%, and 4% of patients, respectively (Table 2). Any grade, grade ≥2, and grade ≥3 ICANS occurred in 22%, 8%, and 4% of patients, respectively. Median time to onset of CRS was 8 days with 4 grade 5 events observed. Median time to onset of ICANS was 9 days with 2 grade 5 events observed. On multivariable analysis, high tumor burden (≥50% bone marrow plasma cells) was associated with higher likelihood of developing grade ≥2 CRS (OR 3.80, 95% CI: 2.05–7.03; Supplementary Table 3). On multivariable analysis, older age ≥70 years (OR 1.98, 95% CI: 1.17–3.37), high plasma cell burden (OR 2.60, 95% CI: 1.35–5.02), hemoglobin <8 g/dL (OR 2.54, 95% CI: 1.37–4.73), and hematopoietic cell transplantation-specific comorbidity index (HCT-CI) ≥ 2 (OR 1.81, 95% CI: 1.20–2.74) were associated with higher risk of developing any grade ICANS (Supplementary Table 4). Non-ICANS neurotoxicity (NINT) was observed in 5% (*n* = 31; Supplementary Table 5). Sixteen patients (2.7%) developed Parkinsonism and 15 patients (2.5%) experienced motor neuron disorders, all of which were cranial nerve palsies, most commonly involving cranial nerve VII (12/15). Median time to onset of Parkinsonism was 10 days (range: 8–21) while median time to onset of cranial nerve palsy was 20 days (range: 17–31). Multivariable analysis for NINT was not performed due to small number of events.

**Table 2:** Safety outcomes in patients who received standard-of-care ciltacabtagene autoleucel.

Event	*N* (%) or median (range)
CRS	
Any grade; grade ≥3	475 (80); 26 (4)
Grade 1	355 (60)
Grade 2	91 (15)
Grade 3	13 (2)
Grade 4	9 (2)
Grade 5	4 (1)
Unknown	3
Median time to onset from CAR-T therapy, days	8 (6–9)
Median duration, days	4 (3–5)
ICANS	
Any grade; grade ≥3	133 (22); 26 (4)
Grade 1	60 (10)
Grade 2	20 (3)
Grade 3	17 (3)
Grade 4	7 (1)
Grade 5	2
Unknown	47
Median time to onset from CAR-T therapy, days	9 (6–12)
Median duration, days	5 (2–10)
NINT	
Any	31 (5)
Parkinsonism	16 (2.7)
Cranial nerve palsies	15 (2.5)
IEC-HS	21 (4)
Clinically significant infection	281 (47)
Bacterial	116 (20)
Viral	194 (33)
Fungal	14 (2)
Other, more than 1 pathogen type	69 (12)
Cytopenias at day 30	145 (24)
Prolonged thrombocytopenia	107 (18)
Prolonged neutropenia	89 (15)
Treatment-related mortality (TRM)	27 (5)
Second primary malignancies (SPM)	27 (5)
SPM excluding non-melanoma skin cancers	18 (3)
Myeloid neoplasms/acute leukemia, myelodysplastic syndrome, T-cell lymphoma	7 (1)

Prolonged cytopenias: defined as nonrecovery of counts by day 30 to the following thresholds among alive patients. Neutrophil recovery: defined as achieving a neutrophil count of >500/µL for 3 consecutive days and the first day is the recovery day. Platelet recovery: defined as platelets 20,000/µL without platelet transfusion in the last 7 days.

*CAR* chimeric antigen receptor, *CRS* cytokine release syndrome, *ICANS* immune effector cell–associated neurotoxicity, *IEC-HS* immune effector cell–associated hemophagocytic lymphohistiocytosis–like syndrome, *NINT* non-ICANS neurotoxicity, *SPM* second primary malignancies, *TRM* treatment-related mortality.

IEC-HS occurred in 4% while clinically significant infections occurred in 47% of patients post CAR-T. Infections were mostly bacterial (20%) or viral (33%), while fungal infections (2%) were less common. Prolonged cytopenias were observed in 24% with neutropenia and thrombocytopenia seen in 15% and 18% of patients at day 30, respectively. SPM occurred in 5%, consisting primarily of nonmelanoma skin cancers. Hematologic malignancies (myelodysplastic syndrome and non-Hodgkin’s lymphoma) occurred in 1%, with no observed malignancies of T-cell origin (Supplementary Table 5).

Ninety-one patients (15%) who received cilta-cel have died by last follow-up: 53 deaths (9%) were attributed to myeloma progression, 27 (5%) were a result of TRM, and death cause was unknown in 11 patients. The cumulative incidence of TRM at 12-months was 5% (95% CI, 3–7). The most common cause of TRM was infection (*n* = 11, including COVID-19, *n* = 1). Other causes of TRM included organ failure (*n* = 8), CRS (*n* = 4), ICANS (*n* = 2), intracranial hemorrhage and other central nervous system pathology (*n* = 2), and SPM (*n* = 2, myelodysplastic syndrome). Two patients had overlapping attribution to CRS and organ failure. No deaths secondary to NINT were observed.

### Response to therapy

**Fig. 1:**
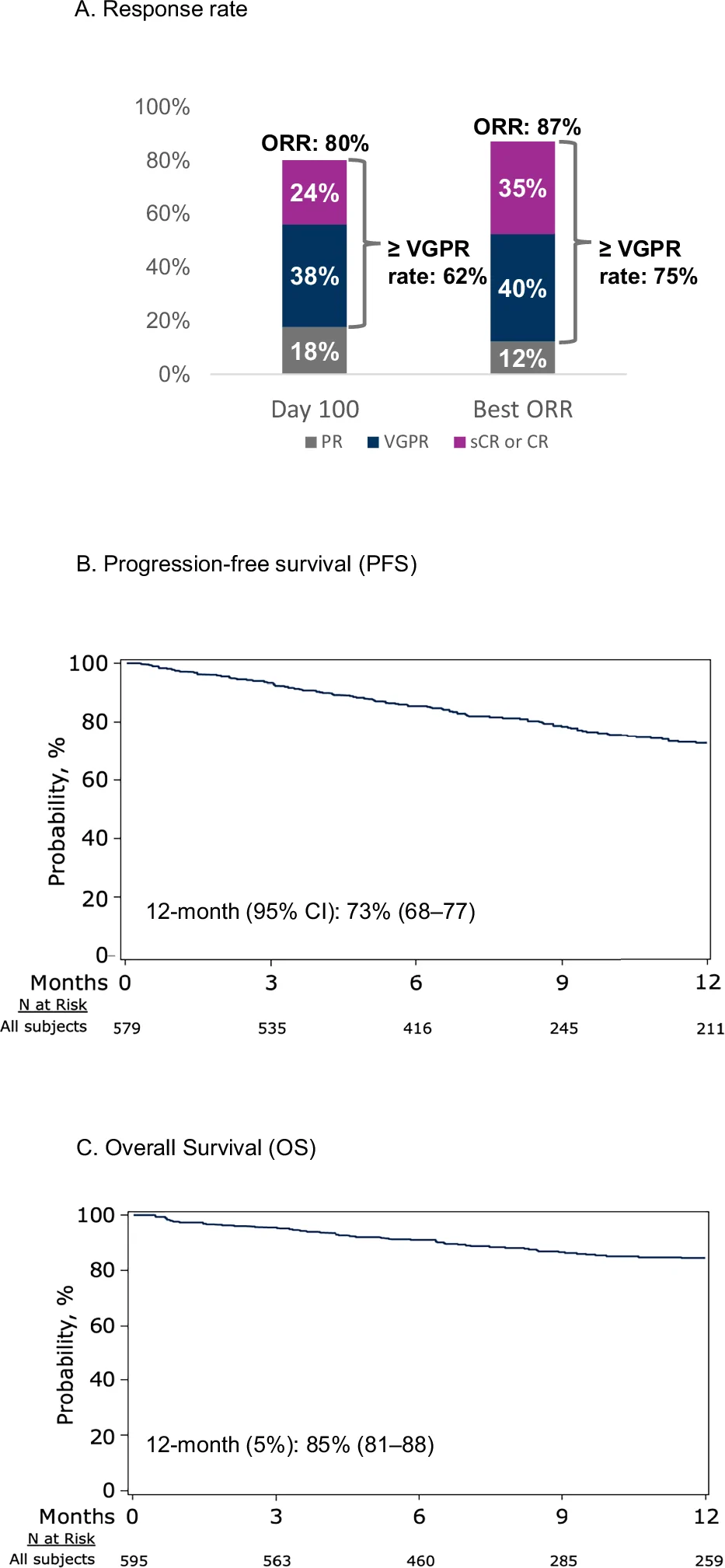
Efficacy with ciltacabtagene autoleucel. **A** Overall response rates by time. **B** Progression-free survival. **C** Overall survival. ORR overall response rate, VGPR very good partial response, CR/sCR complete or stringent complete response, OS overall survivaL, PFS progression-free survival.

The best ORR was 87%, VGPR or better rate was 75%, and CR or better rate was 35% (Fig. 1A). The ORR and CR rates in clinically relevant patient subgroups were as follows: patient age ≥70 years (85% and 34%), ECOG ≥ 2 (74% and 32%), high-risk cytogenetics (88% and 31%), EMD (77% and 40%), ISS stage III (85% and 26%), prior BCMA-directed therapy (70% and 17%), and bendamustine LD regimen (80% and 22%), respectively (Supplementary Table 6). On multivariable analysis, bendamustine LD (OR 0.50, 95% CI: 0.32–0.78, *P* = 0.0025) and Black or African American race (versus white; OR 0.58, 95% CI: 0.37–0.92, *p* = 0.0192) were associated with inferior rates of CR (Supplementary Table 7).

### Survival

**Fig. 2:**
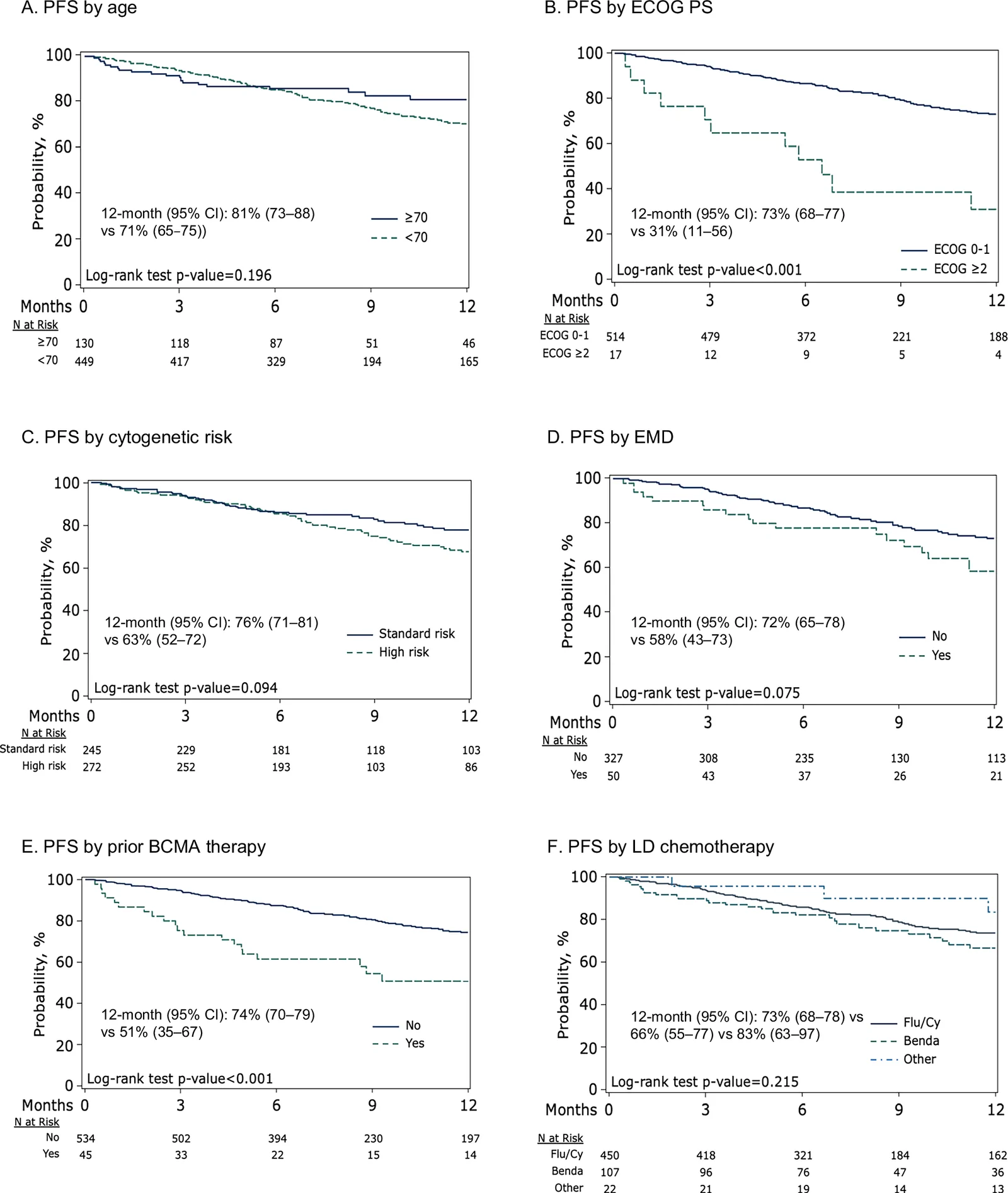
Progression-free survival outcomes with ciltacabtagene autoleucel in patient subgroups. **A** PFS in patients <70 years vs ≥70 years old. **B** PFS in patients with an ECOG PS < 2 vs ≥2. **C** PFS in patients with high-risk vs standard risk cytogenetics. **D** PFS in patients with versus without extramedullary disease. **E** PFS in patients with or without prior BCMA-directed therapy. **F** PFS in patients based on fludarabine and cyclophosphamide vs alternative lymphodepletion regimens. BCMA B-cell maturation antigen, ECOG PS Eastern Cooperative Oncology Group performance status, EMD extramedullary disease, LD lymphodepletion, PFS progression-free survival.

**Fig. 3:**
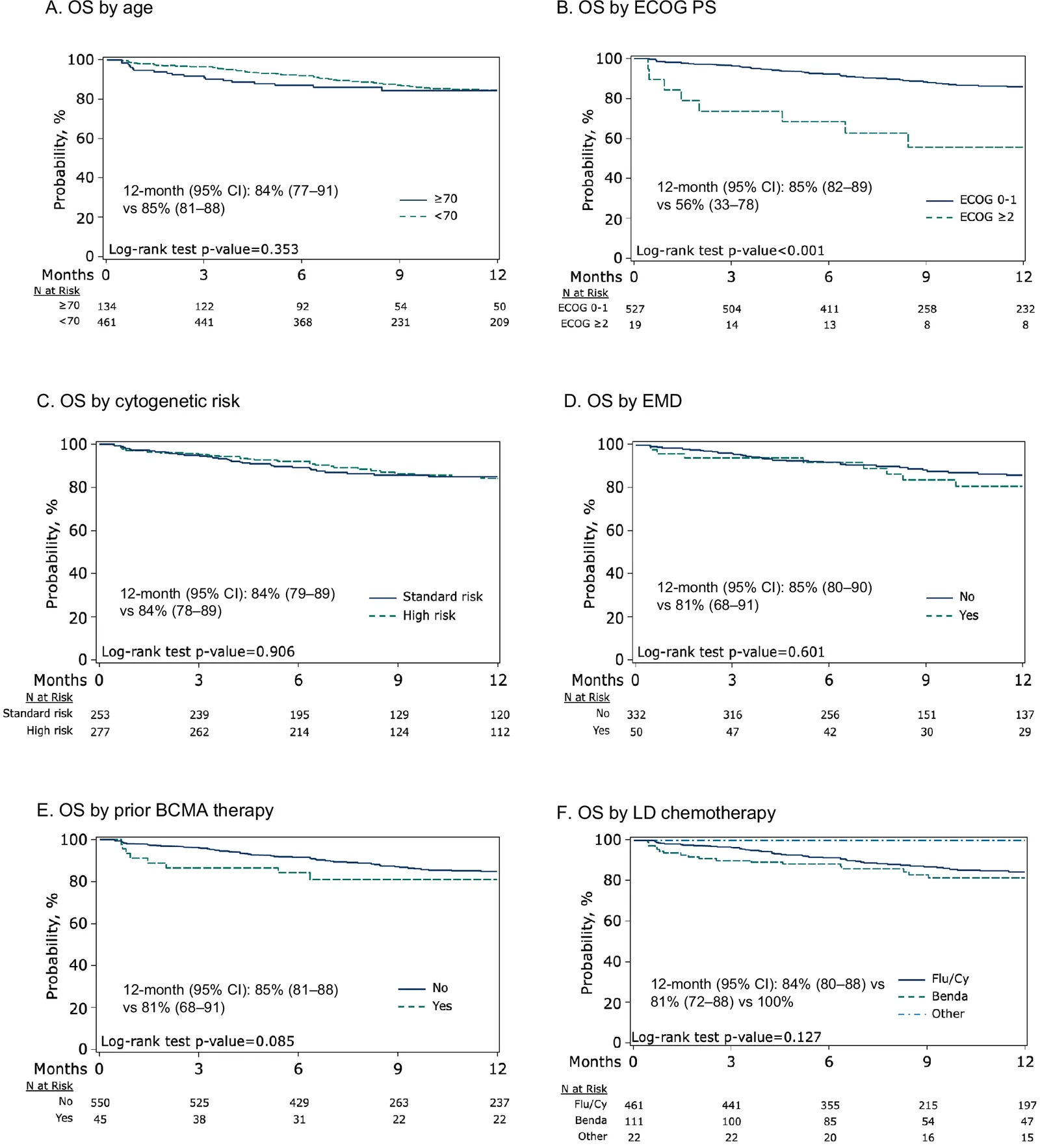
Overall survival outcomes with ciltacabtagene autoleucel in patient subgroups. **A** OS in patients <70 years vs ≥70 years old. **B** OS in patients with an ECOG PS < 2 vs ≥2. **C** OS in patients with high-risk vs standard risk cytogenetics. **D** OS in patients with versus without extramedullary disease. **E** OS in patients with or without prior BCMA-directed therapy. **F** OS in patients based on fludarabine and cyclophosphamide vs alternative lymphodepletion regimens. BCMA B-cell maturation antigen, ECOG Eastern Cooperative Oncology Group performance status, EMD extramedullary disease, LD lymphodepletion, OS overall survival.

Median PFS and OS for the entire cohort (*N* = 595) were not reached. Twelve-month PFS for the entire cohort was 73% (95% CI: 68–77) and 12-month OS was 85% (95% CI: 81–88; Fig. 1B, C). On subgroup analyses (Figs. 2A–F and 3A–F), patients with EMD had a 12-month PFS of 58% compared to 72% in patients without EMD (*P* = 0.08) and a 12-month OS of 81% vs 85% (*P* = 0.60). Patients with high-risk cytogenetics had a 12-month PFS of 63% compared to 76% in patients with standard-risk cytogenetics (*P* = 0.09) and a 12-month OS of 84% vs 84% (*P* = 0.91). The 12-month PFS in patients who had received any prior BCMA therapy (n = 45) was 51% compared to 74% in those without prior BCMA exposure (*P* < 0.001). The 12-month OS was 81% vs 85%, respectively (*P* = 0.09). No significant differences in PFS (12-month PFS: 73% vs 66% vs 83%, *P* = 0.22) and OS (12-month OS: 84% vs 81% vs 100%, *P* = 0.13) were observed between patients who received fludarabine and cyclophosphamide versus bendamustine versus other LD chemotherapy during the fludarabine shortage in the U.S. The 12-month PFS in patients with ISS stage III disease was 50% compared to 58% and 79% in those with Stage II and Stage I disease, respectively (*P* < 0.001). Similarly, 12-month OS was 74%, 77%, and 90% in these groups, respectively (*P* < 0.001).

When evaluating each type of prior BCMA-directed therapy, inferior PFS was observed in patients who received prior belantamab mafodotin and teclistamab. Twelve-month PFS in patients with prior belantamab mafodotin and teclistamab were 50% and 54%, respectively, *P* < 0.001. Twelve-month OS estimates for each of these subgroups were 80% and 86%, respectively, *P* < 0.0001. We also studied the impact of disease status prior to CAR-T infusion. Twelve-month PFS for patients in ≥VGPR vs PR vs SD/PD prior to infusion was 75%, 76%, and 72%, respectively (*P* = 0.39), and the 12-month OS was 91%, 89%, and 83%, respectively (*P* = 0.17; Supplementary Fig. 2).

**Fig. 4:**
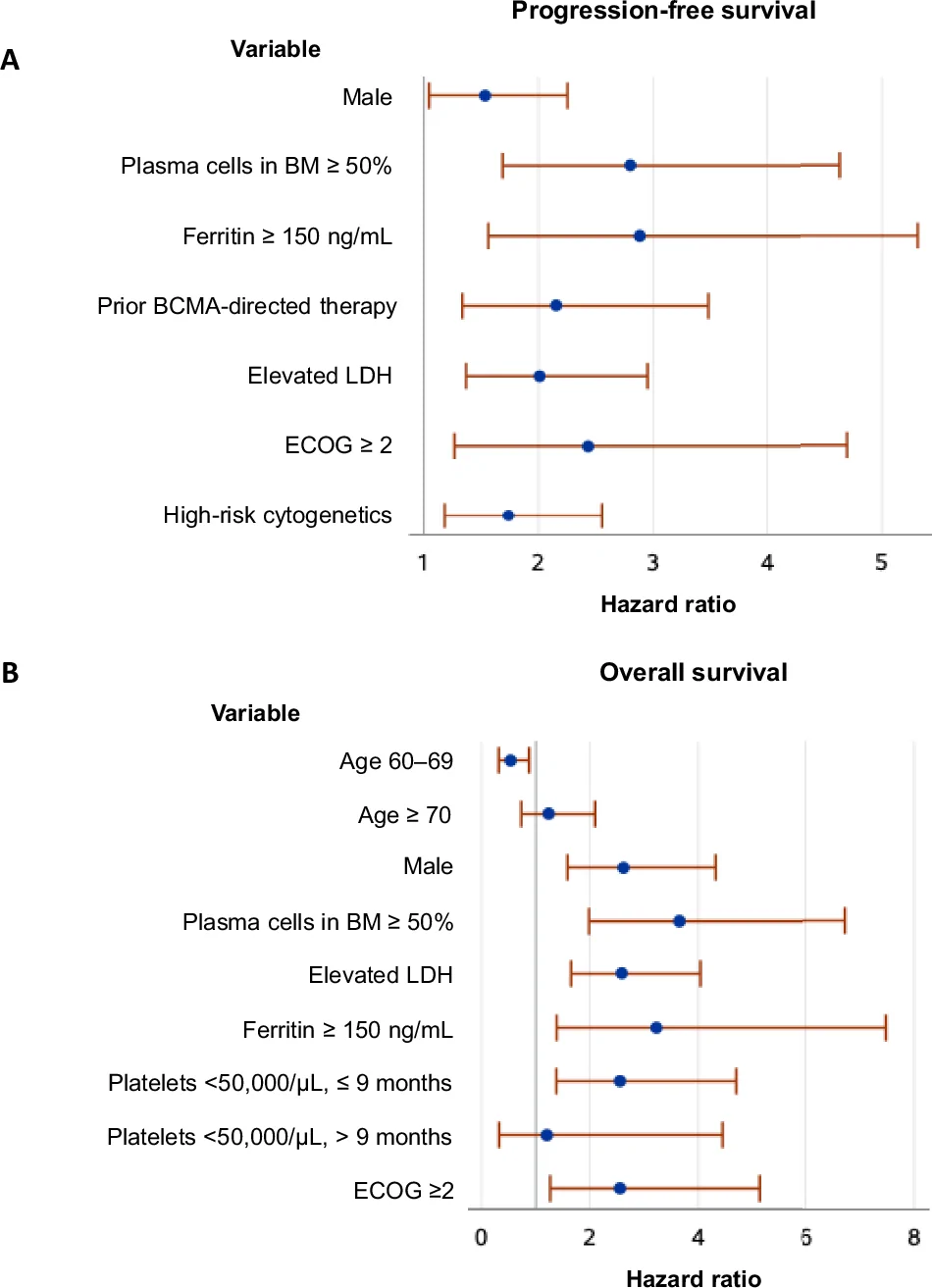
Multivariable Analysis of Progression-Free Survival and Overall Survival in Patients Treated with Ciltacabtagene Autoleucel. Multivariable analyses for **A** progression-free survival and **B** overall survival following ciltacabtagene autoleucel. BCMA B-cell maturation antigen, ECOG PS Eastern Cooperative Oncology Group performance status, BM bone marrow, LDH lactate dehydrogenase, OS overall survival, PFS progression-free survival.

Multivariable analysis identified characteristics associated with inferior PFS and these included male sex, high-risk cytogenetics, prior BCMA-directed therapy, high plasma cell burden, ECOG PS ≥ 2, elevated baseline LDH, and elevated baseline ferritin ≥150 ng/mL (Fig. 4A). Adverse prognostic factors for OS on multivariable analysis included male sex, ECOG PS ≥ 2, elevated baseline LDH, high plasma cell burden, elevated baseline ferritin ≥150 ng/mL, and platelet count <50,000/uL before cilta-cel infusion (Fig. 4B).

## Discussion

To our knowledge, this is the largest study to date reporting real-world outcomes of commercial cilta-cel for RRMM, including 595 patients with a median follow-up of 12 months from infusion. We observed favorable efficacy and safety, despite 70% of patients having clinically significant comorbidities that may have excluded them from CARTITUDE-1 trial participation. Our population was heavily pretreated, with a median of 7 prior lines of therapy, and 8% had previously received BCMA-directed therapy. Despite these high-risk features, SOC cilta-cel achieved favorable outcomes, with an ORR of 87% and 12-month PFS and OS rates of 73% and 85%, respectively. These results are somewhat inferior to those of CARTITUDE-1 (ORR 98%, 12-month PFS 77%, OS 89%), most likely reflecting the high prevalence of clinically significant comorbidities and prior treatment exposures in our population that may have rendered them ineligible for the trial [4–8]. Nevertheless, our findings are consistent with previously published real-world data (ORR 89%, CR rate 35%, 12-month PFS 68%, OS 82%) [11]. It should also be noted that CIBMTR data reporting requires strict adherence to IMWG guidelines and as a result, patients without urine immunofixation confirmation of a complete response would not be classified as such.

Given the large size of our cohort, we were able to conduct robust multivariable analyses to identify predictors of efficacy and survival. Consistent with prior reports, prior BCMA-directed therapy was associated with inferior PFS [18–22]. In our cohort, most patients with prior BCMA exposure had received the ADC belantamab mafodotin, whereas use of bispecific antibodies or CAR-T therapy was uncommon. With the expanding array of BCMA and GPRC5D-directed immunotherapies for RRMM, data to guide treatment selection and sequencing are urgently needed. Prior BCMA exposure has also been associated with lower response rates and PFS following ide-cel [10, 12, 22, 23]. Moreover, a shorter interval from the most recent BCMA-directed therapy has been linked to inferior outcomes, not only in patients receiving commercial CAR-T therapy but also in those treated with anti-BCMA bispecific antibodies [20, 22, 24]. Sequencing novel immunotherapies therefore, remains an area of ongoing clinical investigation and critical importance. Looking forward, integration of molecular biomarkers and genomic prediction models may enable more personalized sequencing strategies, optimizing the use of multiple BCMA- and GPRC5D-directed agents in RRMM [25, 26].

Apart from prior BCMA exposure, poor performance status, high marrow plasma cell burden, and elevated inflammatory markers were independently associated with inferior PFS and OS, consistent with published data on CAR-T therapy and other novel immunotherapies in RRMM [11, 27–29]. High-risk cytogenetics were also linked to inferior PFS but not OS. Another important finding from our study is the prognostic impact of disease status at the time of cilta-cel infusion. Patients achieving VGPR or better at infusion had numerically superior OS compared with those in PR or SD/PD, consistent with results from the KarMMa-3 trial of ide-cel, the CARTITUDE-4 trial of cilta-cel, and other real-world data [7, 11, 30]. Across studies, lower disease burden at infusion has consistently correlated with improved efficacy and safety outcomes, including incidence of NINT [31–35].

Due to a national shortage of fludarabine, many tertiary centers adopted alternative LD regimens, most commonly bendamustine. In our cohort, bendamustine was independently associated with a lower rate of CR or better, as well as a non-statistically inferior PFS and OS compared to fludarabine and cyclophosphamide LD. Prior reports on bendamustine as LD for BCMA CAR-T have been conflicting [36]. A real-world analysis of cilta-cel demonstrated inferior PFS with bendamustine LD compared with fludarabine and cyclophosphamide (3.9 vs 9.1 months) [11]. Collectively, the available data support fludarabine and cyclophosphamide as the preferred LD regimen prior to CAR-T infusion. Residual confounding may partly account for the poorer outcomes observed with bendamustine, along with potential adverse effects on T-cell fitness and the cytokine milieu required for optimal CAR-T expansion and persistence [37].

Despite a substantial proportion of patients presenting with clinically significant comorbidities, baseline cytopenias, and older age, the safety profile of cilta-cel in our cohort was comparable to that reported in the CARTITUDE-1 trial and in prior real-world studies [4, 11]. Grade ≥ 3 CRS and ICANS occurred in 4% of patients, respectively, with these high-grade events more frequently observed in those with high disease burden. The large size of our cohort enabled a multivariable analysis to identify risk factors for grade ≥ 2 CRS and any-grade ICANS with cilta-cel. High marrow plasma cell burden was independently associated with increased risk of both grade ≥ 2 CRS and any-grade ICANS. In addition, older age (≥ 70 years), hemoglobin <8 g/dL, and HCT-CI ≥ 2 were associated with a higher risk of any-grade ICANS. As CAR-T therapy increasingly transitions to outpatient administration, identification of such risk factors may aid in stratifying patients who would benefit from inpatient infusion, intensified bridging chemotherapy, aggressive supportive care management, and prophylactic interventions to mitigate immune-mediated toxicities. Clinically significant infections were observed in 47% of patients, consistent with prior real-world reports (46%) [11]. Most infections were bacterial or viral, as anticipated. Although antimicrobial use was not systematically captured in this registry, and detailed data on intravenous immunoglobulin use was not available compared with CARTITUDE-1, where grades 3 and 4 infections were reported in 20% of patients [4], our findings highlight the clinical importance of intensified antimicrobial prophylaxis and intravenous immunoglobulin support to reduce infection-related morbidity and mortality.

Cilta-cel has been associated with a spectrum of NINT, including nerve palsies and Parkinsonism, some of which may be irreversible and contribute to morbidity and mortality [4]. In our cohort, NINT occurred in 5% of patients, comprising a 2.7% incidence of Parkinsonism and a 2.5% incidence of motor neuron disorders, predominantly seventh cranial nerve palsies. The rates of Parkinsonism and motor neuron disorders were numerically lower in our cohort compared with the CARTITUDE trials (Parkinsonism: 2.7% vs. 5%; motor neuron disorders: 2.5% vs. 10%, including both peripheral neuropathy and cranial nerve palsies) [4, 7, 38]. However, the incidence of Parkinsonism was comparable to that reported in other real-world cilta-cel studies [11]. The most recent CARTITUDE-4 study [7], in which cilta-cel was administered in earlier lines of therapy, reported an even lower incidence of Parkinsonism at 1%. Due to the low frequency of these events in our cohort, we were unable to identify predictors of NINT. The biological underpinnings of these toxicities remain poorly understood.

In CARTITUDE trials, patients who developed cranial nerve palsies demonstrated higher CAR T-cell expansion, suggesting that interventions aimed at reducing CAR T-cell burden, such as high-dose corticosteroids or cytotoxic chemotherapy, may be considered [39]. Most patients with nerve palsies gradually improve and eventually recover with corticosteroids in combination with intravenous immunoglobulin, although recovery often requires several weeks to months [40]. In contrast, standard therapies for Parkinson’s disease have been ineffective in managing CAR T–related Parkinsonism. Some case reports and case series have described symptomatic improvement or resolution with intrathecal chemotherapy or regimens combining cyclophosphamide and corticosteroids [41–43]. Most recently, early mitigation strategies have been adopted in clinical practice, including prophylactic dexamethasone or cyclophosphamide administered at the time of peak absolute lymphocyte count (ALC) expansion (typically between days 10 and 14 post–CAR-T) in patients with elevated ALC, with the goal of reducing the risk of NINT [44]. Emerging single-center experiences have suggested limited utility of dexamethasone prophylaxis in reducing lymphocyte expansion or the incidence of NINT, underscoring the need for further prospective, methodologically robust evaluation and exploration of novel mitigation strategies [45, 46]. However, evidence remains limited, and the optimal intervention, timing, ALC threshold, and potential impact on CAR-T efficacy are not yet established.

More recently, the risk of SPM following CAR-T in MM has gained increasing attention [47–51]. The first confirmed case of a T-cell lymphoma caused by CAR insertion into the TP53 gene during commercial cilta-cel manufacturing was reported by Perica et al. [52]. In our cohort, hematologic SPM occurred in 1% of patients, with no T-cell–derived malignancies observed. However, longer follow-up is required to fully characterize the long-term risk of SPM associated with cilta-cel. We observed a TRM rate of 5%, with an additional 2% of deaths due to unknown causes. This is somewhat lower than the 10% nonrelapse mortality (NRM) reported in the U.S. Multiple Myeloma Immunotherapy Consortium experience and comparable to the 6% NRM observed in CARTITUDE-1 [4, 11]. Infections were the leading cause of death across cohorts, highlighting the need for vigilant monitoring and potentially intensifying prophylactic strategies [53, 54].

The main strengths of our study include, to our knowledge, the largest reported cohort of patients treated with commercial cilta-cel to date. Importantly, we included patients who are typically underrepresented in clinical trials, and the large sample size allowed us to robustly assess and identify predictors of safety and efficacy. These findings not only validate but also extend previously published SOC data. Furthermore, the median follow-up of 1 year allowed us to capture short- and intermediate-term outcomes, although longer follow-up is needed to assess long-term toxicities. The size of our cohort also enabled identification of subgroups with favorable CAR-T outcomes, as well as those with inferior outcomes who may benefit from future consolidative or maintenance therapies after cilta-cel.

Our study has several limitations. Refractoriness to prior therapy after initial response is not captured in CIBMTR forms, limiting our ability to report the proportion of patients who were triple-class refractory or penta-refractory. Information on manufacturing failures and patients who did not receive cilta-cel after apheresis for other reasons is also unavailable. Additionally, measurable residual disease (MRD) status was largely unavailable, preventing MRD-based outcome analyses. Additional limitations include the relatively short follow-up, which may underestimate the incidence of late complications beyond 1 year, particularly SPM and the possibility of under-reporting of NINT. In addition, detailed longitudinal neurologic characterization and progression of neurologic symptoms attributable to NINT could not be reliably assessed within the CIBMTR registry. Other emerging AEs of cilta-cel, most notably immune effector cell-associated enterocolitis (IEC-EC), were not queried of centers in our analysis. Finally, cilta-cel is now available for use in earlier lines of therapy, which may result in better outcomes in future analyses as compared to CARTITUDE-1 or the present study (in which all patients had received at least 4 prior lines of therapy). Despite these limitations, our study provides valuable insights into cilta-cel applicability and outcomes in routine clinical practice.

In conclusion, commercial cilta-cel demonstrated a favorable safety and efficacy profile, leading to deep and durable responses. These outcomes were observed despite most patients having clinically significant comorbidities and extensive prior myeloma therapy, including BCMA-directed treatments. Close monitoring for late complications and intensification of infectious prophylaxis remain critical to minimize NRM. Overall, our findings further support cilta-cel as a highly effective therapeutic option with broad applicability in the SOC management of RRMM patients. As cilta-cel is integrated into earlier lines of treatment, future research should prioritize identifying patients at highest risk for treatment-related toxicities, such as NINT, SPM, and IEC-EC, while advancing preventive and therapeutic strategies to enhance its safety in clinical practice.

## Supplementary information


Supplemental Material
Reproducibility checklist - NOT FOR PUBLICATION


## Data Availability

Analysis datasets from the Center for International Blood and Marrow Transplant Research (CIBMTR) are freely accessible for secondary analysis, with strict measures in place to safeguard participant privacy and protect confidential and proprietary data: (https://cibmtr.org/CIBMTR/Resources/Publicly-Available-Datasets#).
