# Correction: Safety and efficacy of ciltacabtagene autoleucel for relapsed/refractory multiple myeloma: a CIBMTR study

**DOI:** 10.1038/s41408-026-01605-9

**Published:** 2026-08-31

**Authors:** Doris K. Hansen, Danai Dima, Hira Mian, Jakob Devos, Ruta Brazauskas, Temitope Oloyede, Aimaz Afrough, Nausheen Ahmed, Larry D. Anderson, Rahul Banerjee, Jesus G. Berdeja, Aram Bidikian, Binod Dhakal, Ajoy Dias, Yvonne Efebera, Muhammad Salman Faisal, Lohith Gowda, Hamza Hashmi, Abu-Sayeef Mirza, Meera Mohan, Ravi Narra, Ashley E. Rosko, Mark Schroeder, Taiga Nishihori, Heather Landau, Saad Usmani, Marcelo C. Pasquini, Othman S. Akhtar, Surbhi Sidana, Krina K. Patel

**Affiliations:** 1 H. Lee Moffitt Cancer Center & Research Institute, Tampa, FL USA; 2 University of Washington/Fred Hutchinson Cancer Center, Seattle, WA USA; 3 McMaster University/Juravinski Cancer Centre, Hamilton, ON Canada; 4 CIBMTR (Center for International Blood and Marrow Transplant Research), Milwaukee, WI USA; 5 Medical College of Wisconsin, Milwaukee, WI USA; 6 University of Kansas Medical Center, Kansas City, KS USA; 7 UT Southwestern Medical Center/Harold C. Simmons Comprehensive Cancer Center, Dallas, TX USA; 8 Tennessee Oncology, Nashville, TN USA; 9 Yale School of Medicine/Yale Cancer Center, New Haven, CT USA; 10 National Cancer Institute, Bethesda, MD USA; 11 OhioHealth, Columbus, OH USA; 12 OU Health Stephenson Cancer Center, Oklahoma City, OK USA; 13 Memorial Sloan Kettering Cancer Center, New York, NY USA; 14 Ohio State University Comprehensive Cancer Center—James Cancer Hospital, Columbus, OH USA; 15 Washington University School of Medicine/Siteman Cancer Center, St. Louis, MO USA; 16 Cleveland Clinic, Cleveland, OH USA; 17 Stanford University School of Medicine, Stanford, CA USA; 18 The University of Texas MD Anderson Cancer Center, Houston, TX USA

**Keywords:** Cancer therapy, Cancer immunotherapy

Correction to: *Blood Cancer Journal* https://doi.org/10.1038/s41408-026-01496-w, published online 14 April 2026

In this article, the author’s name, ‘Larry D. Anderson, Jr’ has been published erroneously as ‘Larry Anderson’.

The original article has been corrected.

