# Supplementary material for: Safety and efficacy of ciltacabtagene autoleucel for relapsed/refractory multiple myeloma: a CIBMTR study
Source: Blood Cancer J. 2026 Apr 14;16(1):80. doi: 10.1038/s41408-026-01496-w (PMC13194909; doi:10.1038/s41408-026-01496-w)
Supplement: Supplementary file 1 — Supplemental Material [file 41408_2026_1496_MOESM1_ESM.docx]

**SUPPLEMENTARY MATERIAL**

**Supplementary Figures**

1. **Supplementary Fig. 1:** Patient Flow Diagram
2. **Supplementary Fig. 2:** Progression-Free Survival (PFS) and Overall Survival (OS) in patient subgroups by bridging therapy and disease status prior to lymphodepleting chemotherapy

**Supplementary Tables**

1. **Supplementary Table 1:** Type of prior BCMA-directed therapies and efficacy outcomes of ciltacabtagene autoleucel in BCMA-exposed patients
2. **Supplementary Table 2:** Bridging therapy in patients receiving ciltacabtagene autoleucel
3. **Supplementary Table 3:** Multivariable analysis for factors impacting development of cytokine release syndrome (CRS) grade ≥ 2 following ciltacabtagene autoleucel
4. **Supplementary Table 4:** Multivariable analysis for factors impacting development of any grade immune effector cell-associated neurotoxicity syndrome (ICANS) following ciltacabtagene autoleucel
5. **Supplementary Table 5:** Safety outcomes including non-ICANS neurologic toxicity (NINT), second primary malignancies (SPM), and cause of death in patients receiving ciltacabtagene autoleucel
6. **Supplementary Table 6:** Overall response rate (ORR) and complete response (CR) rates in patient subgroups receiving ciltacabtagene autoleucel
7. **Supplementary Table 7:** Multivariable analysis for factors impacting complete response (CR) rates following ciltacabtagene autoleucel

**Supplementary Fig. 1:** Patient Flow Diagram

Study Population: Number of patients treated with commercial ciltacabtagene autoleucel in the United States registered in the CIBMTR database

Infused with ciltacabtagene autoleucel in specification and have at least one follow-up at day 100 or earlier if patient expired prior to day 100

Total Cohort, N=595

Safety, N=595

Responses, N=573 (excluding patients in ≥ CR prior to infusion)

PFS, N=595

OS, N=595

Median follow-up: 12.0 months (range, 1.1-25.4)

*Abbreviations****:*** CIBMTR: Center for International Blood and Marrow Transplant Research. CR: complete response. PFS: progression-free survival. OS: overall survival.

**Supplementary Fig. 2:** PFS and OS in patient subgroups by bridging therapy (A&B) and disease status prior to lymphodepletion (C&D)

**A**

**
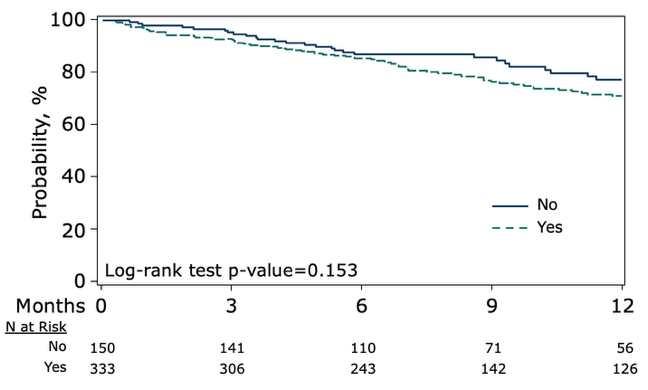
**

12-month PFS (95% CI): 77% (69-85)

vs 71% (65-77)

D

**B**

**
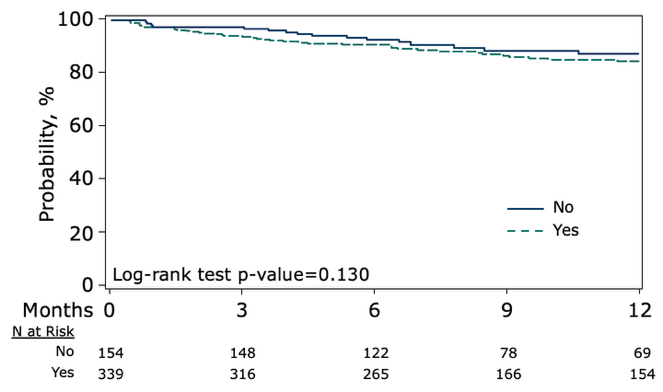
**

12-month OS (95% CI): 87% (81-93)

vs 85% (80-89)

**C**

**
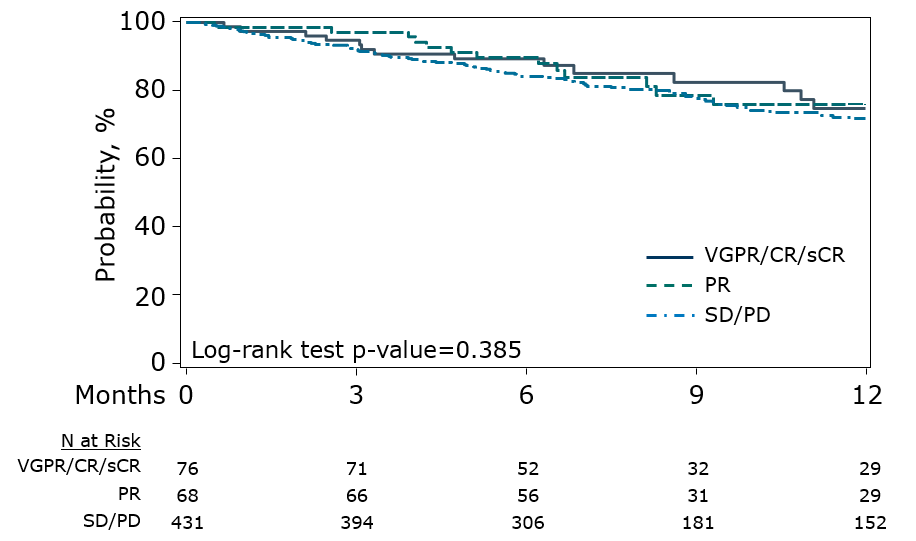
**

12-month PFS (95% CI): 75% (62-86)

vs 76% (63-87) vs 72% (66-77)

**D**

**
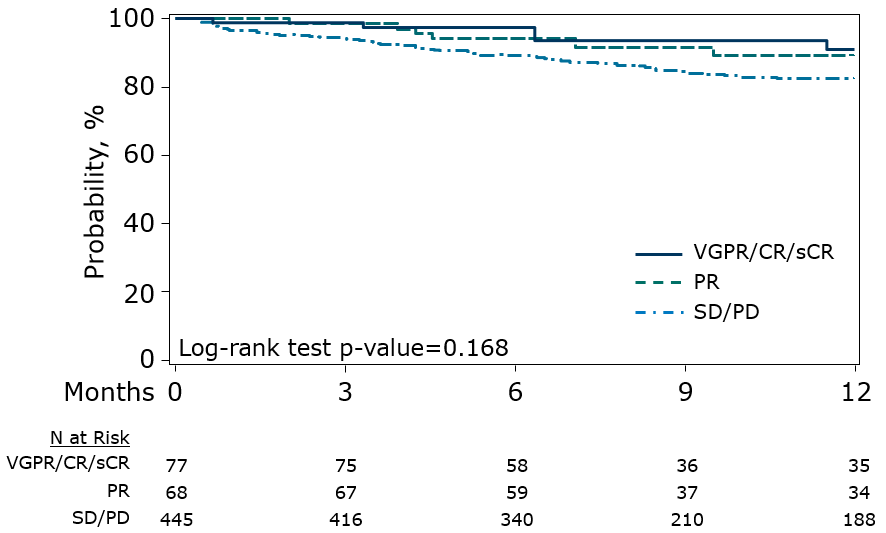
**

12-month (95% CI): 91% (82-97)

vs 89% (79-96) vs 83% (78-86)

*Abbreviations****:*** PFS: progression-free survival, OS: overall survival, CR: complete response, PR: partial response, PD: progressive disease, VGPR: very good partial response, sCR: stringent complete response, SD: stable disease.

**Supplementary Table 1:** Type of prior BCMA therapies and efficacy outcomes of ciltacabtagene autoleucel in BCMA-exposed patients

|  | N or N(%) or median (range) |
| --- | --- |
| **Type of prior BCMA Therapy** | |
| Prior antibody drug conjugate (ADC) (belantamab mafodotin) | 37 (6) |
| Prior BCMA bispecific antibody (teclistamab) | 7 (1) |
| Prior BCMA CAR T-cell therapy* | 3 (0.5) |
| More than one type of prior BCMA therapy | 1 (0.2) |
| **Efficacy outcomes in patients with prior BCMA therapy exposure** | |
| Overall response Rate | 70 |
| Complete Response Rate | 17 |
| 12-month PFS estimate, 95% CI | 51 (35 - 67) |
| 12-month OS estimate, 95% CI | 81 (68 - 91) |

*Abbreviations and definitions****:*** BCMA: B-cell maturation antigen. CAR T-cell therapy: Chimeric antigen receptor T-cell therapy.*Prior CAR-T (n=5) includes bb21217 (n=1), a CD28-based anti-BCMA CAR-T (n=1), an unknown anti-BCMA CAR-T (n=1), a clustered regularly interspaced short palindromic repeats-edited allogeneic anti-BCMA CAR-T (n=1), and anti-G-protein-coupled receptor, class C, group 5, member D (GPRC5D, n=1). PFS: progression-free survival, OS: overall survival.

**Supplementary Table 2:** Bridging therapy in patients receiving ciltacabtagene autoleucel

|  | **N (%)** |
| --- | --- |
| **Bridging Therapy Type** |  |
| Yes | 339 (57) |
| Radiation therapy given as bridging therapy | 33 (10) |
| Systemic therapy given as bridging therapy | 330 (55) |
| Corticosteroids | 168 (50) |
| Carfilzomib | 125 (37) |
| Cyclophosphamide | 118 (35) |
| Pomalidomide | 97 (29) |
| Daratumumab | 70 (21) |
| Etoposide | 56 (17) |
| Bortezomib | 55 (16) |
| Cisplatin | 52 (15) |
| Doxorubicin | 35 (10) |
| Selinexor | 42 (12) |
| Venetoclax | 24 (7) |
| VCd(bortezomib-cyclophosphamide-dexamethasone) | 23 (7) |
| Bendamustine | 15 (4) |
| Isatuximab | 14 (4) |
| Elotuzumab | 13 (4) |
| Ixazomib | 8 (2) |
| Thalidomide | 7 (2) |
| Lenalidomide | 6 (2) |
| KRd (carfilzomib-lenalidomide-dexamethasone) | 6 (2) |
| Doxorubicin | 5 (1) |
| Teclistamab | 5 (1) |
| DVd (daratumumab-bortezomib-dexamethasone) | 4 (1) |
| Cytarabine | 4 (1) |
| Belantamab mafodotin | 4 (1) |
| Carmustine | 1 (0.3) |
| Clarithromycin | 1 (0.3) |
| Talquetamab | 1 (0.3) |
| Other systemic drug(s) | 79 (23) |

**Supplementary Table 3:** Multivariable analysis for cytokine release syndrome (CRS) grade ≥ 2 following ciltacabtagene autoleucel

| **Parameter** | **Category** | **N** | **OR** | **95% CI (lower)** | **95% CI (upper)** | **P-value** |
| --- | --- | --- | --- | --- | --- | --- |
| Plasma cells in bone marrow ≥ 50% prior to infusion | No | 318 | 1.00 | - | - | - |
|  | Yes | 54 | 3.80 | 2.05 | 7.03 | <0.0001 |
|  | Unknown | 220 | 1.21 | 0.77 | 1.89 | 0.41 |

*Abbreviations****:*** CI: confidence interval, CRS: cytokine release syndrome, OR: odds ratio.

**Supplementary Table 4:** Multivariable analysis for any grade immune effector cell-associated neurotoxicity syndrome (ICANS) following ciltacabtagene autoleucel

| **Parameter** | **Category** | **N** | **OR** | **95% CI (lower)** | **95% CI (upper)** | **P-value** |
| --- | --- | --- | --- | --- | --- | --- |
| Age at infusion | < 60 | 203 | 1.00 | - | - | - |
|  | 60 to 69 | 256 | 1.14 | 0.70 | 1.84 | 0.60 |
|  | ≥70 | 134 | 1.98 | 1.17 | 3.37 | 0.01 |
| Plasma cells in bone marrow ≥ 50% prior to infusion | No | 319 | 1.00 | - | - | - |
|  | Yes | 54 | 2.60 | 1.35 | 5.02 | 0.005 |
|  | Unknown | 220 | 1.34 | 0.86 | 2.07 | 0.20 |
| ANC < 750 /uL prior to infusion | No | 555 | 1.00 | - | - | - |
|  | Yes | 21 | 2.05 | 0.77 | 5.50 | 0.15 |
|  | Unknown | 17 | 3.08 | 1.06 | 8.92 | 0.04 |
| Hemoglobin < 8 g/dL prior to infusion | No | 539 | 1.00 | - | - | - |
|  | Yes | 54 | 2.54 | 1.37 | 4.73 | 0.003 |
| HCT-CI | 0-1 | 288 | 1.00 | - | - | - |
|  | ≥2 | 300 | 1.81 | 1.20 | 2.74 | 0.005 |
|  | Unknown | 5 | 2.14 | 0.23 | 19.91 | 0.51 |

*Abbreviations****:*** ANC: absolute neutrophil count, CI: confidence interval, HCT-CI: hematopoietic cell transplantation-specific comorbidity index, ICANS: immune effector cell associated neurotoxicity syndrome, OR: odds ratio.

**Supplementary Table 5:** Safety outcomes including non-ICANS neurologic toxicity (NINT), second primary malignancies (SPM), and cause of death in patients receiving ciltacabtagene autoleucel

|  | **N (%) or median (range)** |
| --- | --- |
| **Type of non-ICANS neurotoxicity (NINT)** |  |
| Cranial nerve palsies | 15 (3) |
| Seventh cranial nerve palsy | 12/15 (80) |
| Other motor neuron disorder | 3/15 (20) |
| Parkinsonism | 16 (2.7) |
| Median time to onset of seventh nerve palsy | 20 (17-31) |
| Median time to onset of Parkinsonism | 10 (8-21) |
| **Second primary malignancies (SPM)** |  |
| Squamous cell skin cancer | 7 (1.2) |
| Myelodysplastic syndrome | 5 (0.8) |
| Genitourinary malignancy | 4 (0.7) |
| Non-Hodgkin’s lymphoma | 2 (0.3) |
| Basal cell skin cancer | 2 (0.3) |
| Gastrointestinal malignancy | 2 (0.3) |
| Lung cancer | 1 (0.2) |
| Breast cancer | 1 (0.2) |
| Melanoma | 1 (0.2) |
| Other malignancy | 2 (0.3) |
| **Cause of death** |  |
| Myeloma-related | 53 (9) |
| Infection | 11 (2) |
| Pulmonary | 4 (0.7) |
| CRS | 4 (0.7) |
| ICANS | 2 (0.3) |
| Multiorgan failure | 2 (0.3) |
| Second Primary Malignancy, MDS | 2 (0.3) |
| Cardiac | 1 (0.2) |
| CNS event/pathology | 1 (0.2) |
| Intracranial hemorrhage | 1 (0.2) |
| Gastrointestinal | 1 (0.2) |
| Unknown | 11 (2) |

*Abbreviations****:*** CRS: cytokine release syndrome, CNS: central nervous system, ICANS: immune effector cell-associated neurotoxicity syndrome, MDS: myelodysplastic syndrome, NINT: non-immune effector cell-associated neurotoxicity syndrome, SPM: second primary malignancies. Two patients had overlapping attribution to CRS and organ failure.

**Supplementary Table 6:** Overall response rate (ORR) and complete response (CR) rates in patient subgroups receiving ciltacabtagene autoleucel

|  | **ORR** | **CR rate** |
| --- | --- | --- |
| **Age**  <70 years  ≥70 years | 88%  85% | 35%  34% |
| **Cytogenetic Risk**  High risk  Standard risk | 88%  89% | 31%  38% |
| **Extramedullary Disease**  Yes  No | 77%  92% | 40%  38% |
| **ISS stage**  Stage I  Stage II  Stage III | 94%  89%  85% | 52%  37%  26% |
| **ECOG PS**  0-1  2 or more | 89%  74% | 36%  32% |
| **Lymphodepletion Therapy**  Fludarabine and cyclophosphamide  Bendamustine  Others | 91%  80%  86% | 41%  22%  27% |
| **Prior BCMA-directed therapy**  No Prior BCMA-directed therapy  Prior BCMA-directed therapy | 91%  70% | 41%  17% |

*Abbreviation and definitions****:*** BCMA: B-cell maturation antigen, CR: complete response, ECOG PS: Eastern Cooperative Oncology Group performance status. *High-risk cytogenetics: Includes del(17p), t(4;14) and t(14;16). ISS: International Staging System, ORR: overall response rate.

**Supplementary Table 7:** Multivariable analysis for factors impacting complete response (CR) rates following ciltacabtagene autoleucel

| **Parameter** | **Category** | **N** | **Odds Ratio** | **95% CI (lower)** | **95% CI (upper)** | **P-value** |
| --- | --- | --- | --- | --- | --- | --- |
| Lymphodepleting chemotherapy | Cyclophosphamide + Fludarabine | 438 | 1.00 | . | . | 0.0098 |
|  | Bendamustine | 106 | 0.50 | 0.32 | 0.78 | 0.0025 |
|  | Other | 22 | 0.83 | 0.42 | 1.62 | 0.5815 |
| Race | White | 434 | 1.00 | . | . | 0.0265 |
|  | Black or African American | 84 | 0.58 | 0.37 | 0.92 | 0.0192 |
|  | Other | 24 | 0.76 | 0.36 | 1.63 | 0.4813 |
|  | Not reported | 24 | 1.70 | 0.90 | 3.24 | 0.1039 |

*Abbreviations****:*** CI: confidence interval.
